# Designing a resilient retail supply network for fresh products under disruption risks

**DOI:** 10.3389/fpubh.2023.1099227

**Published:** 2023-01-25

**Authors:** Zhuyue Li, Peixin Zhao

**Affiliations:** School of Management, Shandong University, Jinan, China

**Keywords:** retail supply chain design, Lagrangian relaxation, disruption risk, resilient supply chain, fresh products

## Abstract

The retail sector supplies the daily fresh products and increasingly plays a key role in the stability and livability of cities. However, public health events such as COVID-19 have caused frequent product shortages in recent years. The risk of fresh product shortages not only causes retailers to lose profits, but also affects the normal life of residents. In this paper, we address the problem of designing a resilient retail supply network for fresh products under the supply disruption risks and propose a bi-objective mixed-integer programming model. This model can help retailers to select suppliers, distribution centers and transportation routes under different scenarios and implement three resilience strategies, namely, priority supply, multiple sourcing and lateral transshipment. We use the ε -constraint method to transform the multi-objective problem into a single objective model and develop a Lagrangian relaxation algorithm to solve the different scale instances. This model is solved for a real-life case of a supermarket to obtain managerial insights. In the case study, this paper shows the set of Pareto fronts with different inventory periods, maximum allowed decay and decay rate. We calculate the expected total cost under targeted disruption scenarios and evaluate the effectiveness of these resilience strategies when implemented concurrently or separately. Our results identify the most critical suppliers and distribution centers that should be fortified. We elaborate that more resilience strategies are not always better and managers need to take appropriate resilience strategies according to their own problems.

## 1. Introduction

In recent years, the operation of fresh products has increasingly become an important magic weapon for retailers to achieve “one strike to win.” In China, large supermarket chains emerged in the 1990s (1) and have become an important channel for the circulation of urban agricultural products (2). According to the statistical results of China's top 100 supermarkets and corporate annual reports, Yonghui superstores ranked the second, with fresh income accounting for 48% of its total retail revenue and Jiajiayue supermarket ranked 7th, with fresh income accounting for 43.7% of its total retail revenue in 2021. However, extreme natural disasters, public health incidents, supplier collusion and blockage of transport channel may cause disruptions in the upstream level of the supply chain, and lead to an inability to meet demand of retailers in the downstream, seriously affecting the resilience of the fresh product supply chain. For example, salmonella contamination in peanut butter involving 361 companies and 3,913 products in 2009 (3). What's more, COVID-19 has hit the upstream and downstream of the agri-food supply chain around the world. In April 2020, at least 6, 225 meat packaging, 834 food-processing plants and 111 farms were affected by COVID-19 cases in the USA (4). In China, Xinfadi (One of the largest wholesale markets for agricultural products) was temporarily closed for 63 days caused by COVID-19 outbreak according to regulation (5). Retail supply chain design research mainly deals with long-term decisions that are costly and almost impossible to reverse (6). Insufficient consideration of disruption events in the supply chain design may lead to serious economic losses. Therefore, considering the complexity and uncertainty of the supply chain network, managers need to design a suitable supply chain to avoid high costs due to supply disruptions.

In order to mitigate the impact of disruption and enhance the resilience of the supply chain, many studies have focused on the design of resilient supply chain (3, 7, 8), which has the ability to prepare, respond, and recover in the face of disruption in advance and can maintain a positive and stable state at acceptable cost and time (9). The design of resilient supply network (10) mainly includes facility location (11), allocation problem, supplier selection (12, 13) and so on. To improve the supply network resilience, researchers mainly adopt proactive and reactive strategies. The proactive strategies deal with the creation of supply chain protections rather than consideration of recovery strategies in supply chain design. Proactive strategies are taken before supply chain disruptions occur. The reactive strategies design supply chain processes and structures which can be adjusted when disruptions occur (14, 15). The proactive strategies mainly include multiple sourcing (16), multiple transportation channels (17), facility dispersion, etc. Reactive strategies include backup supplier (12), safe stock (18), etc. Maharjan and Kato (19) explored and analyzed existing literature on RSCND, particularly focusing on different types of resilience measures used from an analytical modeling perspective. This study found 21 papers on this topic and summarized quantitative resilience measures including multiple sourcing, safety stock, facility redundancy, lateral transshipment, demand coverage and so on. Among them, multiple sourcing (16, 20) is an effective strategy to mitigate the risk of supplier disruption, which can effectively reduce the dependence on a single supplier. Some scholars (21) highlight the impact of inventory control strategies on reducing disruption risks, but some companies who execute lean manufacturing principles may not carry redundant inventory at all, instead accepting disruption risks. Only a few efforts take into consideration lateral transshipment to improve the supply network resilience. Jabbarzadeh et al. (22) proposed a stochastic robust optimization to minimize the total supply chain cost in different disruption scenarios. They determined facility location and lateral transshipment quantities and developed a Lagrangian relaxation algorithm to solve the model.

However, prior research mainly considers how to design supply chain for ordinary products under facilities or transportation disruption (18, 23, 24). Studies considering product perishability are still limited (25–27). It is an important challenge for fresh supply chain that fresh product value deteriorates post-harvest. Some researchers have studied the process of quality degradation for fresh products. Rong et al. (28) proposed a general way that can describe the quality degradation of different food products. In general, exponential decay (29) and linear decay (30, 31) provide the means to analytically product quality decay. For example, Joseph Blackburn (29) assumed that the product value of melons deteriorates exponentially post-harvest until the product is cooled. Li et al. (31) assumed that the product quality declines linearly in the shelf-life.

The literature on resilient perishable product supply chain design mainly includes fresh agri-food supply chain design (27, 32) and blood supply chain design (25, 33). Table 1 lists the most relevant literature of this paper. This paper focuses more on the fresh agri-food supply chain design in the literature. Gholami-Zanjani et al. (32) constructed a bi-objective stochastic programming model to maximize expected profits and minimize emissions, and designed a three-echelon meat green supply chain that integrates product perishability and freshness-dependent product prices. Keizer et al. (27) tracked the product quality of the entire supply network based on the quality decay due to duration and temperature of logistics operations, and constructed a mixed-integer linear programming model to maximize the profit under quality constraints. Goodarzian et al. (34, 35) developed two mathematical models for agri-food supply chain networks considering CO_2_ emissions. Yadav et al. (36) addressed the design of a sustainable multiple-channel fresh food distribution network. Yavari and Zaker (37, 38) studied the resilient supply chain design problems considering the perishable nature of products based on both supply chain and power networks.

**Table 1:** Summary of the reviewed research.

**References**	**Product**	**Perishability**		**Uncertainty**			**Objective**					**Method**	**Solution approach**
		**Yes**	**No**	**Supplier**	**DC**	**Others**	**Profit**	**Distance**	**Emission**	**Time**	**Cost**		
Alikhani et al. (18)	Retail chain			√	√	√					√	MILP	CPLEX
Bottani et al. (3)	Tomato sauce		√	√			√			√		MILP	Ant colony optimization
Gholami-Zanjani et al. (32)	Meat	√		√	√		√		√			MILP	A Monte Carlo optimization approach
Diabat et al. (25)	Blood	√			√	√				√	√	MILP	Lagrangian relaxation and ε -constraint
Sadghiani et al. (39)	Tehran retail chain		√	√		√	√				√	MILP	GAMS/CPLEX
de Keizer et al. (27)	Flowers	√					√					MILP	CPLEX
Heidari-Fathian and Pasandideh (33)	Blood	√		√					√		√	MILP	Lagrangian relaxation and BOF method
Jabbarzadeh et al. (22)	Glass		√	√	√						√	MINLP	Lagrangian relaxation
Cui et al. (13)	General goods		√			√					√	MINLP	Lagrangian relaxation
Yadav et al. (36)	Tomato	√							√	√	√	MILP	ε -constraint and LP metrics method
Benyoucef et al. (44)	General goods		√	√							√	MINLP	ε -constraint, SAA and Lagrangian relaxation
This paper	Fresh products	√		√	√	√				√	√	MILP	Lagrangian relaxation and ε -constraint

To the best of our knowledge, there are very few studies on the resilient fresh product supply chain for retailers. Sadghiani et al. (39) and Alikhani et al. (18) proposed decision models to design retail supply chain network for ordinary products under operational and disruption risks. Yavari et al. (40) developed a resilient perishable product supply chain, but they did not consider the supplier selection.

This paper contributes to the literature on resilient supply chain design in several directions. Firstly, the major contribution of authors' work is the design of the retail supply chain network for fresh products. We put forward a bi-objective formulation to help retailers select suppliers, distribution centers and transportation routes. Secondly, our model takes into consideration priority supply, multiple sourcing and lateral transshipment as resilient strategies. Specially, we calculate the expected total cost under targeted disruption scenarios and evaluate the effectiveness of these resilience strategies when implemented concurrently or separately. Thirdly, we develop ε -constraint method and a Lagrangian relaxation algorithm to solve the model more efficiently.

The rest of this paper is organized as follows. Section 2 formulates the supply chain design problem as a bi-objective mixed-integer linear programming model. In Section 3, we develop the Lagrangian relaxation algorithm to solve the supply chain design model. Section 4 illustrates the effectiveness of the proposed algorithm by numerical examples, investigates the application of the model in a real case study, and presents practical and managerial insights. Finally, we conclude the paper and provide directions for future research.

## 2. Resilient retail supply network model

### 2.1. Model description

**Figure 1:**
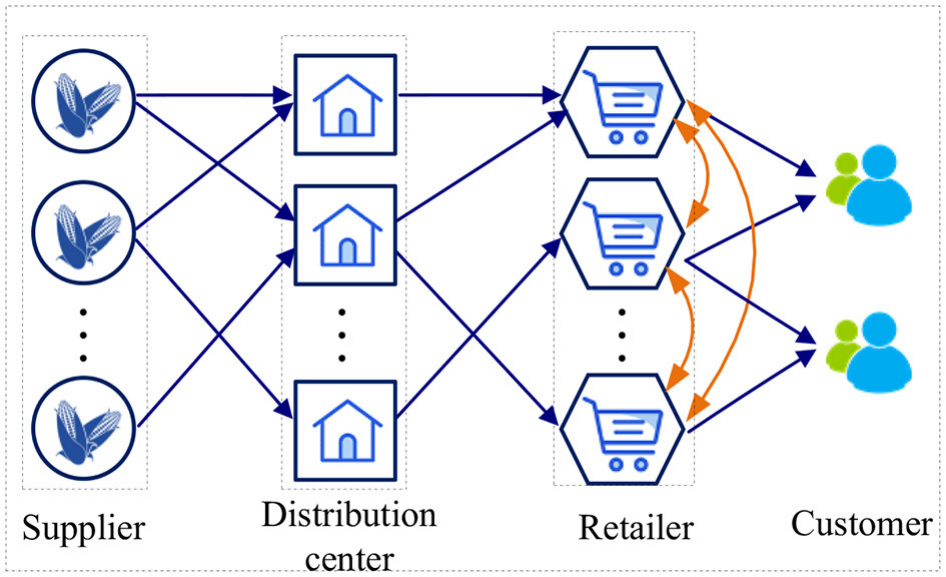
The pattern of supply chain network.

This section designs a resilient supply chain network for perishable products with retailers as the core. The operation of the retail supply chain is shown in Figure 1. Each store operates independently under normal scenarios. However, each store can take into consideration lateral transshipment under disruptions. We consider the problem of multi-product and multi-period supply chain design. The decisions concern the selection of suppliers, the location of Distribution Centers (DCs), the allocation of suppliers to DCs and the allocation of DCs to retailers under different scenarios. We consider a variety of disruption scenarios, such as disruption of suppliers and transportation disruptions between suppliers and DCs, etc.

The basic assumptions are as follows:

1. The retailer opens multiple stores in the market.
2. Each node or arc might be fully or partially disrupted because of damages to roads and infrastructures.
3. Considering the need for sort products, products are not allowed to be distributed directly from the suppliers to the retail stores and only allowed to be distributed from the suppliers through the DCs to the retailer stores.
4. We do not consider the cascading failure caused by supplier and transportation process disrupted.
5. We assume that the product quality declines linearly in the shelf-life.

The existing research pointed out resilience strategies mainly from the perspectives of supplier and inventory. This section does not consider the inventory-related resilience strategies, because keeping extra inventory may reduce the freshness of the product. This section proposes three resilience strategies related to supplier including priority supply, multiple sourcing and lateral transshipment. Priority supply includes three levels. The first level represents general cooperation, which means only the ordering cost. The second level represents friendlier relationships with lower probability of disruption but higher ordering costs. The third level represents the friendliest supply relationship, which has the highest cost, but the probability of disruption is the lowest.

### 2.2. Mathematical model

We present a bi-objective mixed-integer linear programming model to minimize the expected total cost and the lost value. The symbols for formulating the proposed model are defined as follows,

**Table:** 

**Sets and indices**.	
*P*	Set of products, *P* = {*p*|*p* = 1, 2, …, |*P*|};
*H*	Set of suppliers, *H* = {*h*|*h* = 1, 2, …, |*H*|};
*L*	Set of DCs, *L* = {*l*|*l* = 1, 2, …, |*L*|};
*K*	Set of retail stores, *K* = {*k*|*k* = 1, 2, …, |*K*|};
*E*	Set of supply relations, *E* = {*e*|*e* = 1, 2, …, |*E*|};
*S*	Set of disruption scenarios, *S* = {*s*|*s* = 1, 2, …, |*S*|};
*I*	Set of inventory period, *I* = {*i*|*i* = 1, 2, …, |*I*|};
*R*	Set of paths, *R* = {*r*|*r* = 1, 2, …, |*R*|}.
**Parameters**.	
ρ^*s*^	The probability of occurrence for scenario *s*, $\overset{\left|S\right|}{\underset{S=1}{\sum }}\rho^{S}=1$;
$f_{hp}^{e}$	The fixed cost of selecting supplier *h* with supply relation *e* for product *p*;
*f*_*l*_	The fixed cost of selecting DC *l*;
$cw_{hp}^{i}$	In period *i*, selling price of supplier *h* of product *p*;
$cs_{lp}^{i}$	In period *i*, unit processing cost of product *p* in the DC *l*;
$ct_{hlp}^{ir}$	In period *i*, the transportation cost of product *p* from supplier *h* through path *r* to DC *l*;
$ct_{lkp}^{ir}$	In period *i*, the transportation cost of product *p* from DC *l* through path *r* to store *k*;
$ct_{kk^{\prime }p}^{ir}$	In period *i*, the transportation cost of product *p* from store *k* through path *r* to store *k*^′^;
$cap_{l}^{s}$	In scenario *s*, the maximum capacity of DC *l*;
$cap_{hp}^{es}$	The maximum capacity of selecting supplier *h* with supply relation *e* for product *p* under scenario *s*;
$\chi_{hl}^{r}$	The maximum capacity from supplier *h* through path *r* to DC *l*;
$\chi_{lk}^{r}$	The maximum capacity from DC *l* through path *r* to store *k*;
$t_{hl}^{sr}$	Transportation time from supplier *h* through path *r* to DC *l* under scenario *s*;
$t_{lk}^{sr}$	Transportation time from DC *l* through path *r* to store *k* under scenario *s*;
$t_{kk^{\prime }}^{sr}$	Transportation time from store *k* through path *r* to store *k*^′^ under scenario *s*;
*LF*_*p*_	The maximum allowed decay of product *p* for any path;
$D_{kp}^{i}$	In period *i*, the amount of demand in retail store *k* for product *p*;
$pcr_{p}^{k}$	Unit penalty cost of unsatisfied product *p* in store *k*;
*pc*_*p*_	The maximum possible unsatisfied amount of product *p*;
*pk*_*k*_	The maximum lateral transshipment amount of retail store *k*;
α_*p*_	The decay rate of unit time for product *p*.
**Decision variables**.	
$q_{hlp}^{irs}$	The amount of product *p* that is supplied from supplier *h* through path *r* to DC *l* in period *i* under scenario *s*;
$q_{lkp}^{irs}$	The amount of product *p* that is supplied from DC *l* through path *r* to store *k* in period *i* under scenario *s*;
$q_{kk^{\prime }p}^{irs}$	The amount of product *p* that is supplied from store *k* through path *r* to store *k*^′^ in period *i* under scenario *s*;
$X_{hp}^{e}$	1 if supplier *h* with supply relation *e* is selected for product *p*, 0 otherwise;
*V*_*l*_	1 if DC *l* is selected by the SC, 0 otherwise;
$y_{kp}^{is}$	In period*i*, shortage amount of product *p* in retail store *k* under scenario*s*;

(1) $$\begin{array}{l} \min\;F1=\overset{\left|P\right|}{\underset{p=1}{\sum }}\overset{\left|H\right|}{\underset{h=1}{\sum }}\overset{\left|E\right|}{\underset{e=1}{\sum }}X_{hp}^{e}\times f_{hp}^{e}+\overset{\left|L\right|}{\underset{l=1}{\sum }}V_{l}\times f_{l}\\ +\overset{\left|S\right|}{\underset{s=1}{\sum }}\overset{\left|I\right|}{\underset{i=1}{\sum }}\overset{\left|P\right|}{\underset{p=1}{\sum }}(\overset{\left|R\right|}{\underset{r=1}{\sum }}\overset{\left|H\right|}{\underset{h=1}{\sum }}\overset{\left|L\right|}{\underset{l=1}{\sum }}q_{hlp}^{irs}\times \left(ct_{hlp}^{ir}+cw_{hp}^{i}\right)\\ +\overset{\left|R\right|}{\underset{r=1}{\sum }}\overset{\left|K\right|}{\underset{k=1}{\sum }}\overset{\left|L\right|}{\underset{l=1}{\sum }}q_{lkp}^{irs}\times \left(ct_{lkp}^{ir}+cs_{lp}^{i}\right)+\overset{\left|R\right|}{\underset{r=1}{\sum }}\overset{\left|K\right|}{\underset{k=1}{\sum }}\underset{k^{\prime }\in K/\left\{K\right\}}{\sum }q_{kk^{\prime }p}^{irs}\\ \times ct_{kk^{\prime }p}^{ir}+\overset{\left|K\right|}{\underset{k=1}{\sum }}y_{kp}^{is}\times pcr_{p}^{k})\times \rho^{s} \end{array}$$

The objective function (1) aims to minimize the sum of expected cost under different scenarios. The first term of the objective function (1) indicates the fixed cost of supplier selection. The second term of the objective function (1) indicates the fixed cost of DC selection. The third term of the objective function (1) refers to the transportation cost, processing cost, procurement cost, and penalty cost.

(2) $$\begin{array}{l} \min F2=\overset{\left|P\right|}{\underset{p=1}{\sum }}q\left(Atp,\alpha_{p}\right)\\ \;=\overset{\left|P\right|}{\underset{p=1}{\sum }}[\overset{\left|S\right|}{\underset{s=1}{\sum }}\overset{\left|L\right|}{\underset{l=1}{\sum }}(\overset{\left|R\right|}{\underset{r=1}{\sum }}\overset{\left|H\right|}{\underset{h=1}{\sum }}\overset{\left|L\right|}{\underset{l=1}{\sum }}q_{hlp}^{irs}\times t_{hl}^{sr}\\ \;+\overset{\left|R\right|}{\underset{r=1}{\sum }}\overset{\left|L\right|}{\underset{l=1}{\sum }}\overset{\left|K\right|}{\underset{k=1}{\sum }}q_{lkp}^{irs}\times t_{lk}^{sr}\\ \;+\overset{\left|R\right|}{\underset{r=1}{\sum }}\overset{\left|K\right|}{\underset{k=1}{\sum }}\underset{k^{\prime }\in K/\left\{K\right\}}{\sum }q_{kkp}^{irs}\times t_{kk}^{sr})\times \rho^{s}]\times \alpha_{p} \end{array}$$

The objective function (2) minimizes the lost value of the products.

Subject to:

(3) $$\begin{array}{l} \overset{\left|E\right|}{\underset{e=1}{\sum }}X_{hp}^{e}\leq 1\;\forall h\in H,p\in P \end{array}$$

Equation (3) guarantees that only one level of supply relationship could be selected for each established node.

(4) $$\begin{array}{l} \overset{\left|H\right|}{\underset{h=1}{\sum }}\overset{\left|E\right|}{\underset{e=1}{\sum }}X_{hp}^{e}\leq n\;\forall p\in P \end{array}$$

Equation (4) guarantees multiple sourcing.

(5) $$\begin{array}{l} \overset{\left|R\right|}{\underset{r=1}{\sum }}\overset{\left|L\right|}{\underset{l=1}{\sum }}q_{hlp}^{irs}\leq \overset{\left|E\right|}{\underset{e=1}{\sum }}cap_{hp}^{es}\times X_{hp}^{e}\;\forall s\in S,h\in H,i\in I,p\in P \end{array}$$

(6) $$\begin{array}{l} \overset{\left|P\right|}{\underset{p=1}{\sum }}\overset{\left|R\right|}{\underset{r=1}{\sum }}\overset{\left|K\right|}{\underset{k=1}{\sum }}q_{lkp}^{irs}\leq cap_{l}^{s}\times V_{l}\;\forall s\in S,l\in L,i\in I \end{array}$$

Equations (5–6) are capacity constraints of suppliers and DCs.

(7) $$\begin{array}{l} \left(t_{hl}^{sr}\times \alpha_{p}-LF_{p}\right)\times q_{hlp}^{irs}\leq 0\\ \;\forall l\in L,i\in I,s\in S,p\in P,r\in R,h\in H \end{array}$$

(8) $$\begin{array}{l} \left(t_{lk}^{sr}\times \alpha_{p}-LF_{p}\right)\times q_{lkp}^{irs}\leq 0\\ \;\forall l\in L,i\in I,s\in S,p\in P,r\in R,k\in K \end{array}$$

(9) $$\begin{array}{l} \left(t_{kk\prime }^{sr}\times \alpha_{p}-LF_{p}\right)\times q_{kk^{\prime }p}^{irs}\leq 0\\ \;\forall k\in K,s\in S,i\in I,p\in P,r\in R,k^{\prime }\in K/\left\{k\right\} \end{array}$$

Equations (7–9) ensure the quality of the products for any path.

(10) $$\begin{array}{l} \overset{\left|P\right|}{\underset{p=1}{\sum }}q_{hlp}^{irs}\geq \chi_{hl}^{r}\;\forall s\in S,h\in H,l\in L,i\in I,r\in R \end{array}$$

(11) $$\begin{array}{l} \overset{\left|P\right|}{\underset{p=1}{\sum }}q_{lkp}^{irs}\geq \chi_{lk}^{r}\;\forall s\in S,k\in K,l\in L,i\in I,r\in R \end{array}$$

(12) $$\begin{array}{l} \overset{\left|P\right|}{\underset{p=1}{\sum }}q_{kk^{\prime }p}^{irs}\geq \chi_{kk^{\prime }}^{r}\;\forall s\in S,i\in I,r\in R,k\in K,k^{\prime }\in K/\left\{K\right\} \end{array}$$

Equations (10–12) imply that the amount of transportation does not exceed the limit.

(13) $$\begin{array}{l} \overset{\left|L\right|}{\underset{l=1}{\sum }}\overset{\left|R\right|}{\underset{r=1}{\sum }}q_{lkp}^{irs}-\underset{k^{\prime }\in K/\left\{K\right\}}{\sum }\overset{\left|R\right|}{\underset{r=1}{\sum }}q_{kk^{\prime }p}^{irr}+\underset{k^{\prime }\in K/\left\{K\right\}}{\sum }\overset{\left|R\right|}{\underset{r=1}{\sum }}q_{k^{\prime }kp}^{irs}\geq D_{kp}^{i}-y_{kp}^{is}\\ \forall s\in S,k\in K,i\in I,p\in P \end{array}$$

Equation (13) guarantees the amount of supply plus unsatisfied demand is greater than or equal to the customer demand.

(14) $$\begin{array}{l} \underset{k^{\prime }\in K/\left\{K\right\}}{\sum }\overset{\left|R\right|}{\underset{r=1}{\sum }}\overset{\left|P\right|}{\underset{p=1}{\sum }}q_{k^{\prime }kp}^{irs}\leq pk_{k}^{s}\\ \;\forall s\in S,i\in I,k\in K \end{array}$$

(15) $$\begin{array}{l} \underset{k^{\prime }\in K/\left\{K\right\}}{\sum }\overset{\left|R\right|}{\underset{r=1}{\sum }}\overset{\left|P\right|}{\underset{p=1}{\sum }}q_{kk^{\prime }p}^{irs}\leq pk_{k}^{s}\;\forall s\in S,i\in I,k\in K \end{array}$$

Equations (14–15) limit the amount of lateral transshipment to the retailer.

(16) $$\begin{array}{l} \overset{\left|R\right|}{\underset{r=1}{\sum }}\overset{\left|H\right|}{\underset{h=1}{\sum }}q_{hlp}^{irs}=\overset{\left|R\right|}{\underset{r=1}{\sum }}\overset{\left|K\right|}{\underset{k=1}{\sum }}q_{lkp}^{irs}\;\forall s\in S,l\in L,i\in I,p\in P \end{array}$$

Equation (16) denotes that inflows and outflows have to be in balance.

(17) $$\begin{array}{l} \overset{\left|R\right|}{\underset{r=1}{\sum }}\overset{\left|L\right|}{\underset{l=1}{\sum }}q_{lkp}^{irs}\geq \underset{k^{\prime }\in K/\left\{K\right\}}{\sum }\overset{\left|R\right|}{\underset{r=1}{\sum }}q_{kk^{\prime }p}^{irs}\;\forall s\in S,k\in K,i\in I,p\in P \end{array}$$

Equation (17) ensures that the amount of supply is not smaller than the amount of lateral transshipment.

(18) $$\begin{array}{l} y_{kp}^{is}\leq pc_{p}\;\forall s\in S,k\in K,i\in I,p\in P \end{array}$$

Equation (18) limits the unsatisfied demand.

(19) $$\begin{array}{l} y_{kp}^{is},q_{kk\prime p}^{irs},q_{lkp}^{irs},q_{hlp}^{irs}\geq 0\;\forall h\in H,l\in L,k\in K,s\in S,i\in I,r\in R \end{array}$$

(20) $$\begin{array}{l} X_{h}^{e},V_{l}\in \left\{0,1\right\}\;\forall h\in H,l\in L,e\in E \end{array}$$

Equations (19–20) denote non-negativity and binary restrictions of decision variables.

## 3. Solution approach

Multi-objective programming is a part of mathematical programming in which multiple objective functions that should be optimized simultaneously over a feasible set of decisions (25). The weighted-sum method, the ε-constraint method, the goal attainment approach, and meta-heuristics are all commonly used methods to solve multi-objective problems (41). The solution to our bi-objective problem is elaborated in detail in Section 3.1.

The proposed model is a mixed-integer linear programming model whose complexity keeps on rising as the size of the problem increases (42) and commercial software cannot solve the large-scale problems in a reasonable time (33). Therefore, it is necessary to introduce the advanced solution algorithms to solve large-scale problems in a reasonable time. Several algorithms exist for solving large-scale instances such as relaxation, decomposition, and meta-heuristic methods (43). As is well known, many large-scale instances of supply chain design problems have been successfully solved using Lagrangian relaxation. Examples include the work of Benyoucef et al. (44), Cui et al. (13), Heidari-Fathian and Pasandideh (33), and Diabat et al. (25).

### 3.1. Epsilon constraint method

When one objective is more important than the other, ε-constraint method is more appropriate, which can transfer multi-objective problem to single-objective one. What's more, ε-constraint method can also have the advantage of producing non-extreme effective solutions and not needing scale the objective functions to a common scale (45).

In this model, the objective of total cost is more important than that of the lost value. Therefore, we solve the multi-objective problem by ε-constraint method.

The ε-constraint procedure is as follows.

1. The ideal point ($f^{I}=\left(f_{1}^{I},f_{2}^{I}\right)$) is the objective vector minimizing each of the objective functions. That is, $f_{1}^{I}=\min_{X}\left\{f_{1}\left(X\right)\right\}$ and $f_{2}^{I}=\min_{X}\left\{f_{2}\left(X\right)\right\}$. And then, calculate the nadir point ($f^{N}=\left(f_{1}^{N},f_{2}^{N}\right)$). That is, ,.
2. Define $range=f_{2}^{N}-f_{2}^{I}$ and let the interval to Δ. Set $\varepsilon =f_{2}^{N}-\Delta$.
3. Add the constraint *f*_2_ ≤ ε and solve the single-objective problem. The corresponding optimal solution $\left(f_{1}\left(x^{*}\right),f_{2}\left(x^{*}\right)\right)$ is added to the set of Pareto fronts.
4. Set $\varepsilon =f_{2}\left(x^{*}\right)-\Delta$. If $\varepsilon \geq f_{1}^{I}$, then go to Step (3), otherwise, go to Step (5).
5. Obtain the Pareto set.

### 3.2. Lagrangian relaxation

Lagrangian relaxation is an iterative process, which consists of (1) relaxing constraints and introducing them into the objective function; (2) deriving the lower bound by solving the relaxed problem; (3) obtaining a feasible solution as an upper bound; and (4) iterating several times until the difference between the upper bound and the lower bound is very close. The specific process is as follows.

**Step 1. Obtaining the lower bound**.

Considering the Equations (5–6) have both binary variables and continuous variables, this section determines relaxing constraints (5–6) and introduces them into the objective function. Then we calculate the relaxed problem to obtain the lower bound. The relaxed terms are:

(21) $$\begin{array}{l} \overset{\left|S\right|}{\underset{s=1}{\sum }}\overset{\left|H\right|}{\underset{h=1}{\sum }}\overset{\left|I\right|}{\underset{i=1}{\sum }}\overset{\left|P\right|}{\underset{p=1}{\sum }}\lambda_{sh}^{ip}\left(\overset{\left|R\right|}{\underset{r=1}{\sum }}\overset{\left|L\right|}{\underset{l=1}{\sum }}q_{hlp}^{irs}-\overset{\left|E\right|}{\underset{e=1}{\sum }}cap_{hp}^{es}\times X_{hp}^{e}\right) \end{array}$$

(22) $$\begin{array}{l} \overset{\left|S\right|}{\underset{s=1}{\sum }}\overset{\left|L\right|}{\underset{l=1}{\sum }}\overset{\left|I\right|}{\underset{i=1}{\sum }}\lambda_{sl}^{i}\left(\overset{\left|P\right|}{\underset{p=1}{\sum }}\overset{\left|R\right|}{\underset{r=1}{\sum }}\overset{\left|K\right|}{\underset{k=1}{\sum }}\left(q_{lkp}^{irs}-cap_{l}^{s}\times V_{l}\right)\right) \end{array}$$

The resulting relaxed problem is:

(23) $$\begin{array}{l} \min F1+\overset{\left|S\right|}{\underset{s=1}{\sum }}\overset{\left|H\right|}{\underset{h=1}{\sum }}\overset{\left|I\right|}{\underset{i=1}{\sum }}\overset{\left|P\right|}{\underset{p=1}{\sum }}\lambda_{sh}^{ip}\left(\overset{\left|R\right|}{\underset{r=1}{\sum }}\overset{\left|L\right|}{\underset{l=1}{\sum }}q_{hlp}^{irs}-\overset{\left|E\right|}{\underset{e=1}{\sum }}cap_{hp}^{es}\times X_{hp}^{e}\right)\\ \;+\overset{\left|S\right|}{\underset{s=1}{\sum }}\overset{\left|L\right|}{\underset{l=1}{\sum }}\overset{\left|L\right|}{\underset{i=1}{\sum }}\lambda_{sl}^{i}\left(\overset{\left|P\right|}{\underset{p=1}{\sum }}\overset{\left|R\right|}{\underset{r=1}{\sum }}\overset{\left|K\right|}{\underset{k=1}{\sum }}\left(q_{lkp}^{irs}-cap_{l}^{s}\times V_{l}\right)\right) \end{array}$$

Subject to:

(24) $$\begin{array}{l} \overset{\left|P\right|}{\underset{p=1}{\sum }}[\overset{\left|S\right|}{\underset{s=1}{\sum }}\overset{\left|L\right|}{\underset{i=1}{\sum }}(\overset{\left|R\right|}{\underset{r=1}{\sum }}\overset{\left|H\right|}{\underset{h=1}{\sum }}\overset{\left|L\right|}{\underset{l=1}{\sum }}q_{hlp}^{irs}\times t_{hl}^{sr}+\overset{\left|R\right|}{\underset{r=1}{\sum }}\overset{\left|L\right|}{\underset{l=1}{\sum }}\overset{\left|K\right|}{\underset{k=1}{\sum }}q_{llp}^{irs}\\ \;\times t_{lk}^{sr}+\overset{\left|R\right|}{\underset{r=1}{\sum }}\overset{\left|K\right|}{\underset{k=1}{\sum }}\underset{k^{\prime }\in K/\left\{K\right\}}{\sum }q_{kk^{i}p}^{irs}\times t_{kk^{\prime }}^{sr})\times \rho^{s}]\times \alpha \leq \varepsilon \end{array}$$

Equations (3–4) and (7–20).

The relaxed problem is divided into two sub-problems. The first sub-question is:

(25) $$\begin{array}{l} F_{sub1}=\overset{\left|P\right|}{\underset{p=1}{\sum }}\overset{\left|H\right|}{\underset{h=1}{\sum }}\overset{\left|E\right|}{\underset{e=1}{\sum }}X_{hp}^{e}\times \left(f_{hp}^{e}-\overset{\left|S\right|}{\underset{s=1}{\sum }}\overset{\left|I\right|}{\underset{i=1}{\sum }}\lambda_{sh}^{ip}\times cap_{hp}^{es}\right)\\ \;+\overset{\left|L\right|}{\underset{l=1}{\sum }}V_{l}\times \left(f_{l}-\overset{\left|L\right|}{\underset{l=1}{\sum }}\overset{\left|I\right|}{\underset{i=1}{\sum }}\lambda_{sl}^{i}\times cap_{l}^{s}\right) \end{array}$$

Subject to:

Equations (3–4) and (20).

The second sub-question is:

(26) $$\begin{array}{l} \begin{array}{c} F_{sub2}=\overset{\left|S\right|}{\underset{s=1}{\sum }}\overset{\left|I\right|}{\underset{i=1}{\sum }}\overset{\left|P\right|}{\underset{p=1}{\sum }}\left(\overset{\left|R\right|}{\underset{r=1}{\sum }}\overset{\left|K\right|}{\underset{k=1}{\sum }}\underset{k^{\prime }\in K/\left\{K\right\}}{\sum }q_{kk^{\prime }p}^{irs}\times ct_{kk\prime p}^{ir}+\overset{\left|K\right|}{\underset{k=1}{\sum }}y_{kp}^{is}\times pcr_{p}^{k}\right)\times \rho^{s}\\ \;+\overset{\left|S\right|}{\underset{s=1}{\sum }}\overset{\left|H\right|}{\underset{h=1}{\sum }}\overset{\left|I\right|}{\underset{i=1}{\sum }}\overset{\left|P\right|}{\underset{p=1}{\sum }}\overset{\left|R\right|}{\underset{r=1}{\sum }}\overset{\left|L\right|}{\underset{l=1}{\sum }}q_{hlp}^{irs}\times \left[\left(ct_{hlp}^{ir}+cw_{hp}^{i}\right)\times \rho^{s}+\lambda_{sh}^{ip}\right]\\ \;+\overset{\left|S\right|}{\underset{s=1}{\sum }}\overset{\left|L\right|}{\underset{l=1}{\sum }}\overset{\left|I\right|}{\underset{i=1}{\sum }}\overset{\left|P\right|}{\underset{p=1}{\sum }}\overset{\left|R\right|}{\underset{r=1}{\sum }}\overset{\left|K\right|}{\underset{k=1}{\sum }}q_{lkp}^{irs}\times \left[\left(ct_{lkp}^{ir}+cs_{lp}^{i}\right)\times \rho^{s}+\lambda_{sl}^{i}\right] \end{array} \end{array}$$

Subject to:

Equations (7–19) and (25).

By analyzing the *F*_*sub*1_, we can obtain that:

(27) $$\begin{array}{l} X_{hp}^{e}=1,\text{then }X_{hp}^{e}\times \left(f_{hp}^{e}-\overset{\left|S\right|}{\underset{s=1}{\sum }}\overset{\left|I\right|}{\underset{i=1}{\sum }}\lambda_{sh}^{ip}\times cap_{hp}^{es}\right)\\ =f_{hp}^{e}-\overset{\left|S\right|}{\underset{s=1}{\sum }}\overset{\left|I\right|}{\underset{i=1}{\sum }}\lambda_{sh}^{ip}\times cap_{hp}^{es} \end{array}$$

(28) $$\begin{array}{l} X_{hp}^{e}=0,\text{then }X_{hp}^{e}\times \left(f_{hp}^{e}-\overset{\left|S\right|}{\underset{s=1}{\sum }}\overset{\left|I\right|}{\underset{i=1}{\sum }}\lambda_{sh}^{ip}\times cap_{hp}^{es}\right)=0; \end{array}$$

(29) $$\begin{array}{l} V_{l}=1;\text{then }V_{l}\times \left(f_{l}-\overset{\left|L\right|}{\underset{l=1}{\sum }}\overset{\left|I\right|}{\underset{i=1}{\sum }}\lambda_{sl}^{i}\times cap_{l}^{s}\right)=f_{l}-\overset{\left|L\right|}{\underset{l=1}{\sum }}\overset{\left|I\right|}{\underset{i=1}{\sum }}\lambda_{sl}^{i}\times cap_{l}^{s} \end{array}$$

(30) $$\begin{array}{l} V_{l}=0,\text{then }V_{l}\times \left(f_{l}-\overset{\left|L\right|}{\underset{l=1}{\sum }}\overset{\left|I\right|}{\underset{i=1}{\sum }}\lambda_{sl}^{i}\times cap_{l}^{s}\right)=0 \end{array}$$

We design the Algorithm 1 to solve the minimum value of the *F*_*sub*1_ and the pseudo-code can be summarized as follows:

**Algorithm 1:** The second subproblem is a linear programming problem that can be solved by the solver CPLEX.

The minimum value of the *F*_*sub*1_. **Input** $cap_{l}^{s}$, $cap_{hp}^{es}$, *f*_*l*_, $f_{hp}^{e}$, $\lambda_{sl}^{i}$, $\lambda_{sh}^{ip}$ $F_{hp}^{e}\leftarrow f_{hp}^{e}-\overset{\left|S\right|}{\underset{s=1}{\sum }}\overset{\left|I\right|}{\underset{i=1}{\sum }}\lambda_{sh}^{ip}\times cap_{hp}^{es}$, $F_{l}\leftarrow f_{l}-\overset{\left|S\right|}{\underset{s=1}{\sum }}\overset{\left|I\right|}{\underset{i=1}{\sum }}\lambda_{sl}^{i}\times cap_{l}^{s}$ **Initialize** $X_{hp}^{e}$ = 0, *V*_*l*_ = 0 **for** *p* = 1, …, |*P*| **for** *h* = 1, …, |*H*| **if** $\underset{e\in E}{\min}F_{hp}^{e}<0$, **then** $X_{hp}^{e}=1$ (for the corresponding supply relation *e*) **for** *p* = 1, …, |*P*| **if** $\underset{e\in E}{sum}\left(\underset{h\in H}{sum}\left(X_{hp}^{e}\right)\right)<n$ **for** *h* = 1, …, |*H*| *Fa*_*h*_← $\underset{e\in E}{\min}\left(F_{hp}^{e}\right)$ $\underset{h\in H}{sort}\left(Fa_{h},ascend\right)$, select the top *n* supplier, and then $X_{hp}^{e}=1$ **for** *l* = 1, …, |*L*| **If** min *F*_*l*_ < 0, **then** *V*_*l*_ = 1

**Step 2. Obtaining the upper bound**.

Any feasible solution is an upper bound of the original problem. The solution obtained by the step (1) may be infeasible in the original problem and can be modified to derive a new feasible solution. We keep the solution of some variables and find the feasible solutions of other variables under the minimum expected total cost.

**Step 3. Update the Lagrange multiplier**.

For each the Lagrange multiplier λ, we can find the corresponding upper and lower bounds. In each iteration, the values of the Lagrange multipliers are updated, which updates the values of the upper and lower bounds. The values of the Lagrange multipliers are updated as follows:

λ is updated by the subgradient method. Δ^*t*^ is the step size at iteration t,

(31) $$\begin{array}{l} \Delta_{1}^{t}=\frac{\alpha^{t}\left(UB-L^{t}\right)}{\overset{\left|S\right|}{\underset{s=1}{\sum }}\overset{\left|H\right|}{\underset{h=1}{\sum }}\overset{\left|I\right|}{\underset{i=1}{\sum }}\overset{\left|P\right|}{\underset{p=1}{\sum }}{\left(\overset{\left|R\right|}{\underset{r=1}{\sum }}\overset{\left|L\right|}{\underset{l=1}{\sum }}q_{hlp}^{irs}-\overset{\left|E\right|}{\underset{e=1}{\sum }}cap_{hp}^{es}\times X_{hp}^{e}\right)}^{2}} \end{array}$$

(32) $$\begin{array}{l} \Delta_{2}^{t}=\frac{\alpha^{t}\left(UB-L^{t}\right)}{\overset{\left|S\right|}{\underset{s=1}{\sum }}\overset{\left|L\right|}{\underset{l=1}{\sum }}\overset{\left|I\right|}{\underset{i=1}{\sum }}{\left(\overset{\left|P\right|}{\underset{p=1}{\sum }}\overset{\left|R\right|}{\underset{r=1}{\sum }}\overset{\left|K\right|}{\underset{k=1}{\sum }}\left(q_{lkp}^{irs}-cap_{l}^{s}\times V_{l}\right)\right)}^{2}} \end{array}$$

(33) $$\begin{array}{l} \lambda_{sh}^{ip,t+1}=\max\left\{0,\lambda_{sh}^{ip,t}+\Delta_{1}^{t}\left(\overset{\left|R\right|}{\underset{r=1}{\sum }}\overset{\left|L\right|}{\underset{l=1}{\sum }}q_{hlp}^{irs}-\overset{\left|E\right|}{\underset{e=1}{\sum }}cap_{hp}^{es}\times X_{hp}^{e}\right)\right\} \end{array}$$

(34) $$\begin{array}{l} \lambda_{sl}^{i,t+1}=\max\left\{0,\lambda_{sl}^{i,t}+\Delta_{2}^{t}\left(\overset{\left|P\right|}{\underset{p=1}{\sum }}\overset{\left|R\right|}{\underset{r=1}{\sum }}\overset{\left|K\right|}{\underset{k=1}{\sum }}\left(q_{lkp}^{irs}-cap_{l}^{s}\times V_{l}\right)\right)\right\}, \end{array}$$

where *UB* is the best obtained upper bound, and *L*^*t*^ is the current obtained lower bound at iteration t. The iteration stops when the upper and lower bounds are sufficiently close.

## 4. Numerical example

### 4.1. Performances of solution procedure

In this section, numerical examples are given to verify the effectiveness of the proposed solution. All computations were implemented in MATLAB R2020a for Windows ×64 on the laptop with an Intel i7-1260P CPU and 16 GB RAM.

We test proposed algorithms on the numerical examples in different scales. In order to simplify, the potential size of each instance is defined as follows: (|*H*|, |*L*|, |*K*|, |*E*|, |*I*|, |*R*|, |*P*|, |*S*|); e.g., (6, 3, 45, 3, 5, 3, 2, 20) represents six potential suppliers, three potential distribution centers, forty-five stores, three potential relations, five potential periods, three potential paths, two products, and twenty scenarios. This section generates a number of numerical experiments to analyze the performances of solution procedure. The ranges of the parameters are shown in Table 2. And Table 3 analyzes the performance comparison of the results obtained by the Lagrangian relaxation algorithm and the solver CPLEX.

**Table 2:** The ranges of the parameters.

**Parameter**	**Range**	**Parameter**	**Range**
$f_{hp}^{e}$	[200–900] ¥	$\chi_{hl}^{sr}$	[0–200] kg
*f*_*l*_	[600–700] ¥	$\chi_{lk}^{r}$	[0–200] kg
$cw_{hp}^{i}$	[90–190] ¥ /100 kg	$t_{hl}^{sr}$	[0.25–2.2] h
$cs_{lp}^{i}$	[50–100] ¥ /100 kg	$t_{lk}^{sr}$	[0.27–2.29] h
$ct_{hlp}^{ir}$	[0.1–53] ¥/100 kg	*LF*_*p*_	0–0.2
$ct_{lkp}^{ir}$	[0.5–37.5] ¥/100 kg	$D_{kp}^{i}$	(2–5) kg
*pk*_*k*_	0.5 kg	$pcr_{p}^{k}$	[0–500] kg
$cap_{l}^{s}$	[0–5, 000] kg	*pc*_*p*_	[0–500] kg
$cap_{hp}^{es}$	[0–900] kg	α_*p*_	0–0.04

**Table 3:** The performance comparison of the results.

**Set**	**Objective**		**Gap**	**Objective**	
	**Lagrangian relaxation**	**CPLEX**		**Lagrangian relaxation**	**CPLEX**
(3, 2, 5, 3, 1, 1, 1, 1)	2, 247	2, 247	0%	3.3 s	4.8 s
(6, 3, 45, 3, 5, 3, 2, 20)	2, 65, 032	2, 65, 032	0%	5.2 s	8.4 s
(6, 3, 45, 3, 10, 1, 2, 20)	5, 30, 328	5, 30, 327	0%	4.4 s	9.4 s
(8, 4, 50, 3, 10, 1, 3, 20)	8, 53, 208	8, 53, 204	0%	6.0 s	13.4 s
(6, 3, 50, 3, 10, 3, 2, 30)	5, 94, 262	5, 94, 262	0%	6.8 s	15.1 s
(6, 3, 115, 3, 3, 3, 2, 20)	4, 13, 427	4, 13, 427	0%	17.3 s	51.8 s
(6, 3, 50, 3, 3, 3, 9, 30)	8, 18, 670	8, 18, 670	0%	25.9 s	244.4 s
(6, 3, 60, 3, 3, 3, 9, 30)	9, 96, 993	9, 96, 992	0%	384.0 s	869.4 s
(6, 3, 115, 3, 3, 3, 9, 20)	1, 914, 697	1, 914, 697	0%	51.0 s	278.4 s
(8, 4, 80, 3, 3, 3, 9, 27)	1, 610, 084	1, 610, 064	0.001%	182.7 s	160.0 s
(8, 4, 60, 3, 2, 3, 9, 30)	6, 97, 842	6, 97, 858	0.002%	342.5 s	1, 046.3 s
(8, 4, 55, 3, 3, 3, 9, 30)	9, 47, 795	9, 47, 815	0.002%	193.5 s	2, 233.5 s
(8, 4, 50, 3, 3, 3, 9, 30)	8, 51, 570	8, 51, 570	0%	298.4 s	1, 746 s
(6, 3, 150, 3, 3, 3, 9, 20)	2, 515, 861	2, 515, 862	0%	138.9 s	1, 175 s
(6, 3, 115, 3, 3, 3, 9, 25)	1, 915, 135	1, 915, 113	0.001%	92.7 s	393 s

We used the Lagrangian relaxation algorithm and the solver CPLEX to address 15 problems with the minimum expected total cost. As shown in Table 3, relaxing these constraints can reduce the computational time. The advantages of the algorithm become more significant with the solving scale gradually increasing. The solver CPLEX can effectively solve the small- and medium-scale instances in a reasonable length of time, but it takes a considerable amount of time to solve large-scale instances. For example, the thirteenth instance requires 1,746 s using CPLEX and 298 s using Lagrangian relaxation. On the other hand, the gap does not exceed 0.002% in all tested instances, which confirms the effectiveness of Lagrangian relaxation algorithm.

### 4.2. Case study

**Figure 2:**
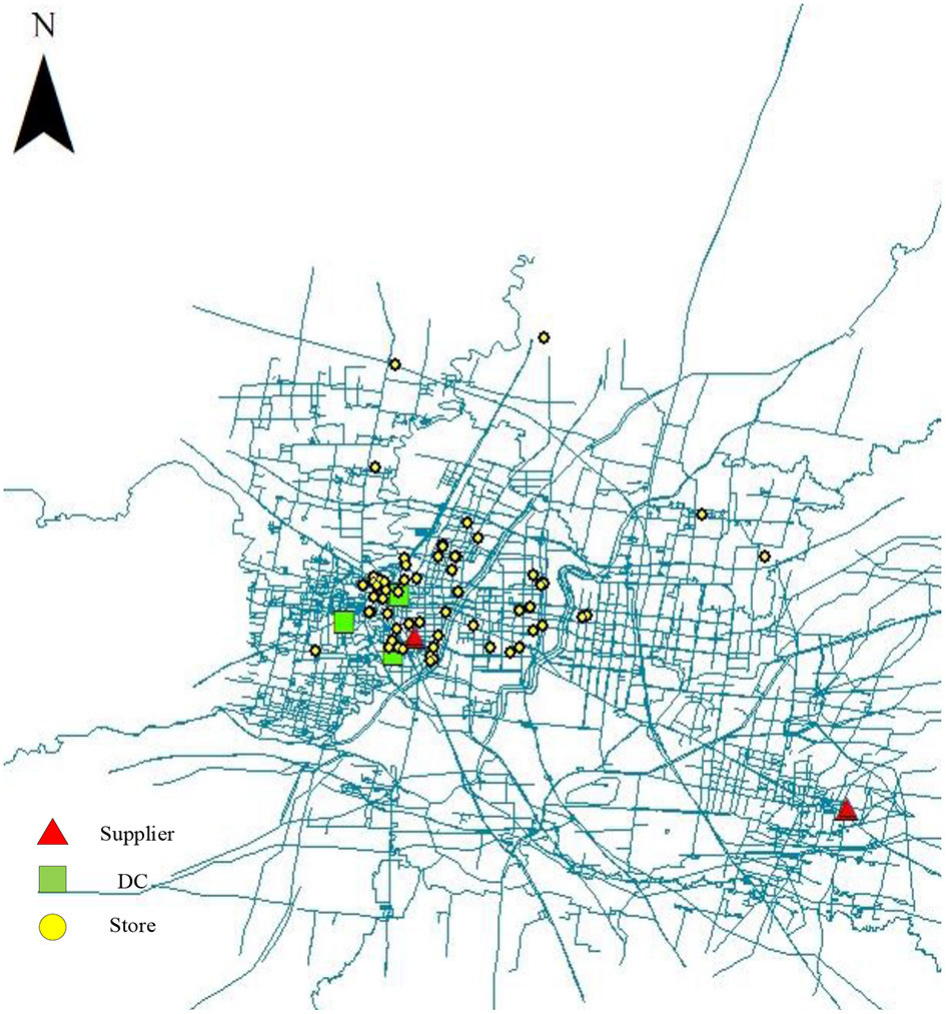
The locations of stores, suppliers and DCs.

Our model is illustrated on a real case in Shandong province, China. The retailer would like to design a resilient novel supply chain against natural disasters, COVID-19 pandemic and online shopping. We use some of that data to design a supply chain. Among them, 58 stores in the retailer need to purchase two products from six potential suppliers and three potential distribution centers. The distance and time from suppliers to the distribution center are determined according to the geographical location. The locations of stores, suppliers and the distribution centers are shown in Figure 2. And the other parameters are shown in Table 2.

Zhalechian et al. (46) calculated the total number of scenarios (*TNS*) with *n* nodes and *d* types of disruptions as: $TNS={\left(1+d\right)}^{\frac{n^{2}+n}{2}}$.

So, it is necessary to reduce the *TNS* to make the problem more tractable. In this paper, we refer to the method based on maximum likelihood sampling in the reference (18, 23, 46), and select the first 20 scenarios with the highest probabilities and normalize the probabilities of 20 scenarios.

#### 4.2.1. Sensitivity analysis

**Figure 3:**
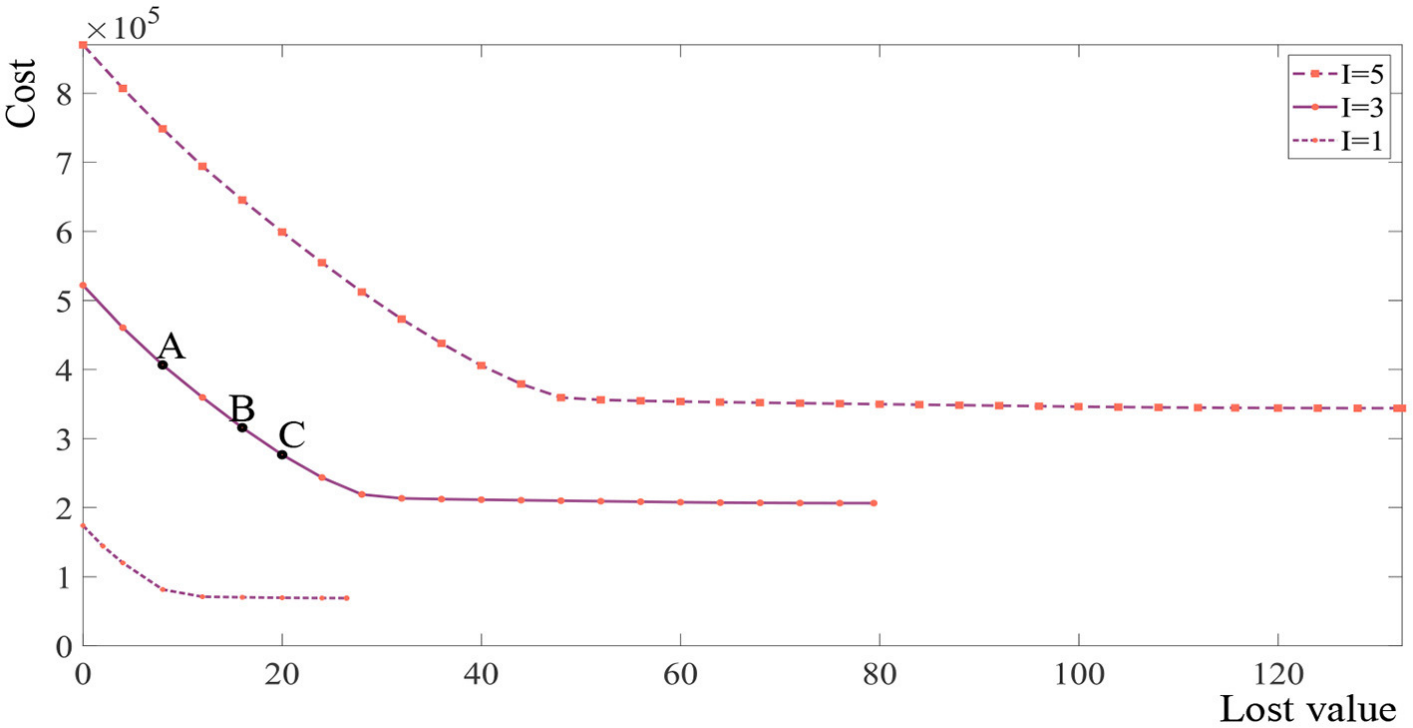
Distribution of the Pareto front of the instance.

This section obtains the set of Pareto fronts of *I* = 5, *I* = 3, and *I* = 1 as shown in Figure 3. It can be seen that the shapes of the Pareto fronts are similar and not straight lines in all three examples. Accordingly, every solution located on the curves presents a non-dominated solution. For example, when *I* = 3, the points A, B, and C present the non-dominated solutions. It can be seen that when the lost value is small, reducing the unit lost value requires a significant increase in the total expected costs and when the lost value is large, reducing the unit lost value requires a slight increase in the total expected costs.

Reducing the expected cost and the deterioration of fresh products can improve the core competitiveness of enterprises. However, this is impossible because some conflicts among objectives exist. The enterprise managers can trade off the transportation time and the total expected cost according to the actual situation of the supply chain.

**Figure 4:**
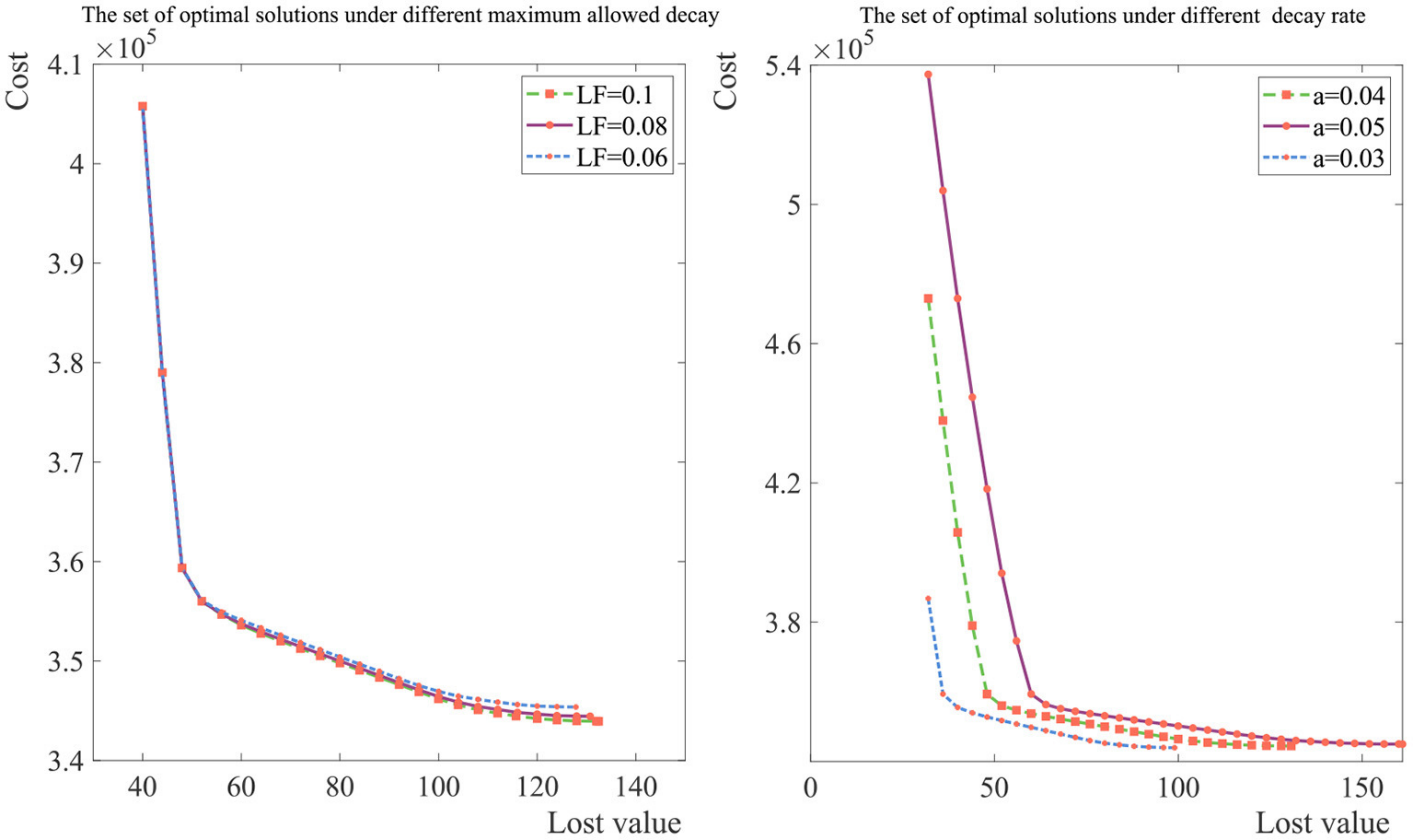
Distribution of the Pareto front under the different maximum allowed decay and decay rate.

In Figure 4, we analyze the Pareto front under different maximum allowed decay and different decay rate. The expected cost is smaller as the maximum allowed decay becomes larger and the expected cost is smaller as the decay rate becomes slower, which is consistent with our experience in life. What's more, it can be seen that inflection point of the curve is not the same for different product, and the managers should redesign the supply chain for new products rather than directly adopting the design results of other products.

**Figure 5:**
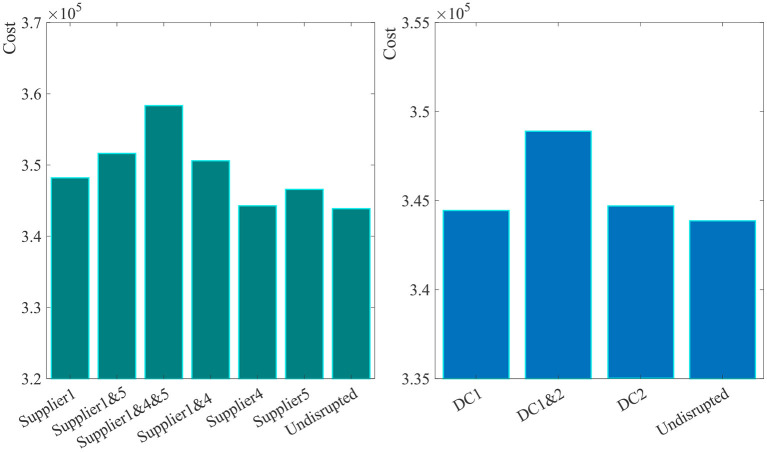
The expected total cost under different disruption scenarios.

In addition, this section analyzes the expected cost of the supply chain under different disruption scenarios. According to the proposed model, the supplier one, supplier four, supplier five, DC one and DC two are selected under the normal operation of supply chain. We analyze the expected total cost when these facilities fail respectively or simultaneously as shown in Figure 5. It can be seen that the expected total cost when supplier one fails is higher than that when supplier four or supplier five fails. If it is difficult for retailers to reinforce the relationships with all suppliers simultaneously, it is more important for the retailer to strengthen its relationship with supplier one than with other suppliers. What's more, the expected total cost when DC two fails is higher than that when DC one fails. Similarly, if it is difficult for the retailer to reinforce the relationships with all DCs simultaneously, it is more important for the retailer to strengthen relationships with DC two than with other DCS.

#### 4.2.2. The effect of resilience strategies

**Figure 6:**
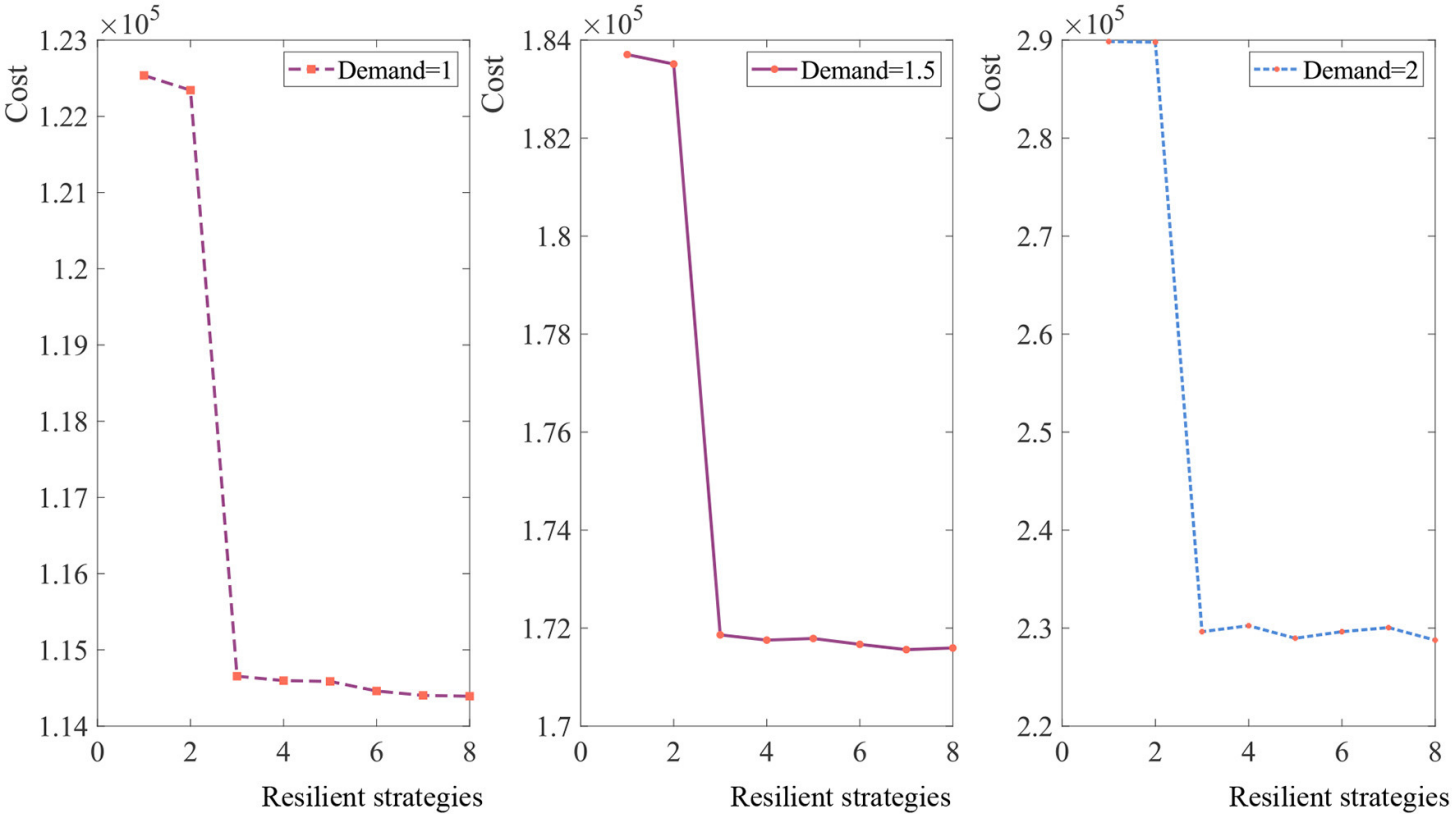
The expected total cost under different resilient strategies.

To study the influence of each resilience strategy, we solve the problem while controlling for the other strategies, as shown in Figure 6. The x-axis represents the resilience strategies. (1) Represents taking no resilience strategies; (2) represents lateral transshipment; (3) represents multiple sourcing; (4) represents priority supply; (5) represents priority supply and multiple sourcing; (6) represents multiple sourcing and lateral transshipment; (7) represents lateral transshipment and priority supply; and (8) represents taking three resilience strategies simultaneously. In Figure 6, the trends of three curves are similar but not identical under different demands. Priority supply and multiple sourcing are more effective in reducing the expected total cost than lateral transshipment.

To show the complementary effects of resilience strategies, we simulate various combinations of resilience strategies adopted concurrently. Obviously, not every combination can be useful. But in most cases, taking two resilience strategies simultaneously can more effective than taking one. For example, when the demand is 1 kg, the expected cost when taking multiple sourcing and priority supply strategies is ¥114586, the expected cost when taking multiple sourcing and lateral transshipment strategies is ¥114461, and the expected cost when lateral transshipment and priority supply strategies implemented is ¥114, 402. In these cases, the expected cost of taking two resilience strategies is lower than taking one strategy separately.

**Figure 7:**
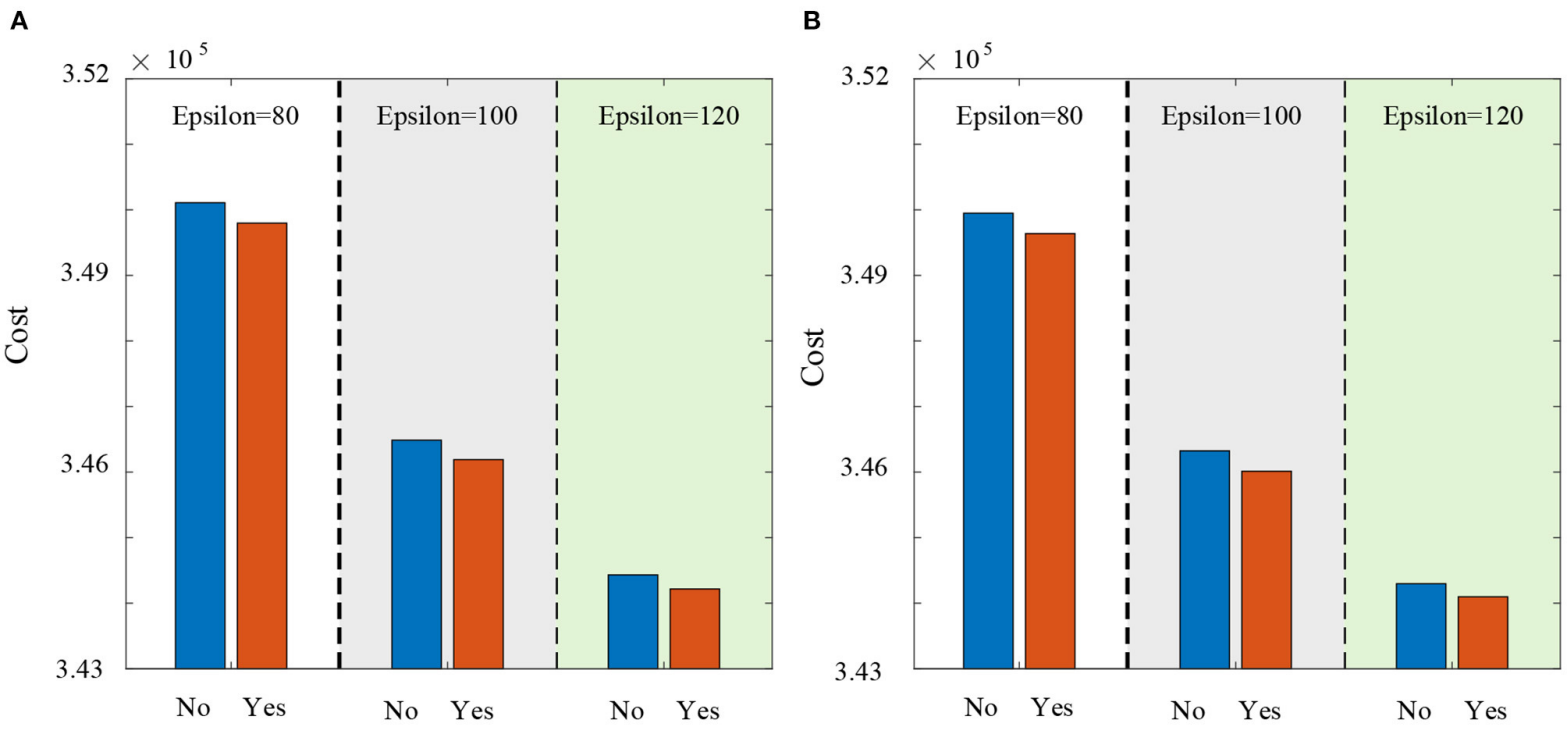
The influence of lateral transshipment strategy. **(A)** The influence of lateral transshipment strategy under the supply chain disruption risks. **(B)** The influence of lateral transshipment strategy under the normal operation of supply chain.

What's more, we simulate the influence of lateral transshipment strategy. Figures 7A, B respectively elaborate the influence of lateral transshipment strategy under the supply chain disruption risks and under the normal operation of supply chain. It has been shown that the lateral transshipment strategy is useful to reduce the supply chain expected cost whether supply chain disruption risks are considered or not. For example, the expected cost is 3,46,486 when the lateral transshipment strategy is not implemented and the expected cost of taking lateral transshipment strategy is 3,46,190 in Figure 7A.

#### 4.2.3. Theoretical, managerial and policy implications

The results provide managerial implications for supply chain practitioners and offering theoretical insight to fill gaps in the literature.

Firstly, this paper designs a resilient retail supply network for fresh products and contributes to the theoretical development of supply chain risk management issues in retailers. Secondly, it can be seen that the shapes of the Pareto fronts are not the same for different products and the Pareto front has significant links to the manager's decision. Therefore, the managers should redesign the supply chain for new products rather than directly adopting the design results of old products. Thirdly, if we only consider one resilience strategy, priority supply and multiple sourcing can reduce the expected total cost more effectively than lateral transshipment in our case study. The lateral transshipment strategy is useful but less effective to reduce the supply chain expected cost whether supply chain disruption risks are considered or not. Fourthly, in most cases, taking two resilience strategies simultaneously can more effective than taking one. However, not every combination can be useful. Therefore, more resilience strategies are not always better and managers need to take appropriate resilience strategies according to their own problems. Finally, in order to reduce the disruption risks, governments can provide policy support to retailers, such as policy for enterprises building relationships with suppliers.

## 5. Conclusions

This paper presents a model that takes the possibility of supply chain disruption into the design of retail supply chain for fresh product. We formulate a multi-product and multi-period bi-objective mixed-integer programming model with priority supply, multiple sourcing and lateral transshipment resilience strategies. Considering the characteristics of fresh products, the two objectives are to minimize the expected total cost and lost value of products during transportation. We transfer multi-objective problem to single-objective one by ε-constraint method and develop Lagrangian relaxation to solve the problem. We evaluate the Lagrangian relaxation method by solving 15 problems with various sizes. It is notable that when the size of the problems increases, the efficiency of the Lagrangian relaxation algorithm also increases. In the case study, we solve the model under different inventory periods, disruption scenarios, maximum allowed decay, decay rate and resilience strategies, and obtain the managerial insights.

As for future research, the research can be extended in a number of directions. Firstly, accounting for imprecise scenario-based data, robust optimization may be an important direction. Secondly, this paper studies the retail supply chain network design problem under determined demand. It can be an interesting direction to consider the retail supply chain design problem under supply and demand uncertainty. Finally, this study does not consider the cascading failure caused by supplier and transportation process disrupted. However, many epidemics are highly infectious, and one facility failure may trigger other facilities failures. The study of supply chain design problem considering the cascading failures can be valuable.

## Data availability statement

The original contributions presented in the study are included in the article/supplementary material, further inquiries can be directed to the corresponding author.

## Author contributions

ZL: conceptualization, methodology, formal analysis, investigation, writing—original draft, and writing—review and editing. PZ: software, validation, formal analysis, data curation, writing—original draft, funding acquisition, and writing—review and editing. All authors contributed to the article and approved the submitted version.
